# Extracellular High Mobility Group Box 1 Plays a Role in the Effect of Bone Marrow Mononuclear Cell Transplantation for Heart Failure

**DOI:** 10.1371/journal.pone.0076908

**Published:** 2013-10-18

**Authors:** Masahiro Kaneko, Yasunori Shintani, Takuya Narita, Chiho Ikebe, Nobuko Tano, Kenichi Yamahara, Satsuki Fukushima, Steven R. Coppen, Ken Suzuki

**Affiliations:** 1 William Harvey Research Institute, Barts and The London School of Medicine and Dentistry, Queen Mary, University of London, London, United Kingdom; 2 Department of Regenerative Medicine and Tissue Engineering, National Cerebral and Cardiovascular Center, Suita, Osaka, Japan; 3 Cardiovascular Surgery, Osaka University Graduate School of Medicine, Suita, Osaka, Japan; Cardiological Center, Italy

## Abstract

Transplantation of unfractionated bone marrow mononuclear cells (BMCs) repairs and/or regenerates the damaged myocardium allegedly due to secretion from surviving BMCs (paracrine effect). However, donor cell survival after transplantation is known to be markedly poor. This discrepancy led us to hypothesize that dead donor BMCs might also contribute to the therapeutic benefits from BMC transplantation. High mobility group box 1 (HMGB1) is a nuclear protein that stabilizes nucleosomes, and also acts as a multi-functional cytokine when released from damaged cells. We thus studied the role of extracellular HMGB1 in the effect of BMC transplantation for heart failure. Four weeks after coronary artery ligation in female rats, syngeneic male BMCs (or PBS only as control) were intramyocardially injected with/without anti-HMGB1 antibody or control IgG. One hour after injection, ELISA showed that circulating extracellular HMGB1 levels were elevated after BMC transplantation compared to the PBS injection. Quantitative donor cell survival assessed by PCR for male-specific *sry* gene at days 3 and 28 was similarly poor. Echocardiography and catheterization showed enhanced cardiac function after BMC transplantation compared to PBS injection at day 28, while this effect was abolished by antibody-neutralization of HMGB1. BMC transplantation reduced post-infarction fibrosis, improved neovascularization, and increased proliferation, while all these effects in repairing the failing myocardium were eliminated by HMGB1-inhibition. Furthermore, BMC transplantation drove the macrophage polarization towards alternatively-activated, anti-inflammatory M2 macrophages in the heart at day 3, while this was abolished by HMGB1-inhibition. Quantitative RT-PCR showed that BMC transplantation upregulated expression of an anti-inflammatory cytokine *IL-10* in the heart at day 3 compared to PBS injection. In contrast, neutralizing HMGB1 by antibody-treatment suppressed this anti-inflammatory expression. These data suggest that extracellular HMGB1 contributes to the effect of BMC transplantation to recover the damaged myocardium by favorably modulating innate immunity in heart failure.

## Introduction

Transplantation of stem or progenitor cells is an emerging approach to repair and/or regenerate damaged myocardium undergoing adverse ventricular remodeling. Unfractionated bone marrow mononuclear cells (BMCs) contain several kinds of stem/progenitor cells and are the most frequently used donor cell type in clinical cell therapy to the heart [1]. The therapeutic effect of BMC transplantation in not only acute myocardial infarction (MI) but also post-MI chronic heart failure (ischemic cardiomyopathy) has been confirmed in animal and human studies [1]–[3]. Because injected BMCs do not vigorously differentiate to functioning cardiomyocytes or vascular cells *in vivo*, the major mechanism of the therapeutic effects is proposed to be their secretion of cytokines, chemokines and growth factors that help repair of the damaged myocardium suffering post-MI adverse remodeling [1]–[3]. However, the precise mechanism of this “paracrine effect” remains uncertain.

Interestingly, cardiac function recovery by BMC transplantation occurs despite of markedly poor donor cell survival [1], [3], [4]. It has also been shown that active secretion from BMCs is less extensive compared to other donor cell types [5], [6]. It was also reported that injection of extract of dead BMCs by freeze-thaw cycles induces the similar therapeutic effect to injection of living BMCs [7]. These findings led us to hypothesize that dead donor BMCs might be a supplementary or alternative source of the paracrine mediators, which could contribute to the repair of the failing myocardium.

High-mobility group box 1 (HMGB1) was initially identified as a nuclear protein that regulates transcriptional factors to stabilize the nucleosome [8]. This molecule is also known to be actively secreted from activated inflammatory cells and also passively released from dead cells [9]–[11]. Extracellular HMGB1 induces and intensifies inflammation in most cases, while it can also operate to attenuate inflammation and enhance the healing of damaged tissues, according to the form/amount of HMGB1 and nature of the tissues [9]–[12]. In the heart, there is increasing evidence that extracellular HMGB1 attenuates myocardial damage and induces recovery/regeneration [13]–[18], though there are contradicting reports [19], [20]. We have demonstrated that HMGB1 administration achieved the similar benefits to the BMC-mediated paracrine effects, including decreased fibrosis, increased vascular formation, attenuated cardiomyocyte hypertrophy, and attenuated inflammation in a rat ischemic cardiomyopathy model [17]. It has also been reported that extracellular HMGB1 augments tissue regeneration through activating endogenous progenitor cells [15], [21].

Collectively, these data formed a hypothesis that extracellular HMGB1 released from dead donor cells contributes to the paracrine effect of BMC transplantation to repair the post-MI failing myocardium and to improve cardiac performance.

## Materials and Methods

### Ethics Statement

All studies were performed with the approval of the UK Home Office (Project Licence Number: 70/7254). The investigation conforms to the Principles of Laboratory Animal Care formulated by the National Society for Medical Research and the Guide for the Care and Use of Laboratory Animals (US NIH Publication, 1996). All animal surgery was performed under inhalation anesthesia of isoflurane and administration of buprenorphine hydrochloride was made just after surgery to reduce postoperative pain, and all efforts were made to minimize suffering. Surgical procedures, cardiac function measurement, and sample analyses were performed in a blinded manner.

### BMC Collection

Bone marrow was isolated from both femurs and tibias of male Lewis rats (150–200 g; Charles River, UK), from which BMCs (mononuclear cells) were purified by Ficoll-Paque gradient centrifugation (GE Healthcare) as previously described [3]. Flow cytometry analysis (FACSAria, BD Biosciences) using monoclonal anti-rat CD34 (Santa-Cruz) and anti-rat CD45 (BD Pharmingen) antibodies showed that 4.6±1.7% of the BMCs were positive for CD34 and 75.5±4.3% were positive for CD45 (**Figure S1**). To trace the injected cells, BMCs were labeled with CM-DiI (Molecular Probes) before transplantation according to the company’s protocol. The viability of donor BMCs just before injection measured by trypan blue staining was 97.1±0.6% (n = 11 animals).

### Assessment of Cardiac Function

Cardiac function and dimensions pre and post treatment were measured by using echocardiography (Vevo−770, VisualSonics) as previously described [3], [17]. Diastolic and systolic LV endocardial areas at the papillary muscle level were measured from parasternal short-axis views, from which LV fractional area change (LVFAC) was calculated. Post treatment hemodynamics parameters were measured by catheterization (SRP−320/PVAN3.2, Millar Instruments and Chart 5 software, ADInstruments) as described previously [22].

### Generation of Ischemic Cardiomyopathy and Cell Transplantation in Rat

Female Lewis rats (150–200 g, Charles River) underwent left coronary artery ligation as described previously [3], [17], [23], [24]. Four weeks later, the animals that showed appropriate cardiac dysfunction (LVFAC 22–32%; base line in intact rats = 61.6±1.7% [n = 5]) by echocardiography were chosen and randomly assigned to 4 treatment groups; intramyocardial injection of 1×10^7^ syngeneic male BMCs (BMC group), injection of BMCs with 50 µg anti-HMGB1 neutralizing antibody (Medical & Biological Laboratories; AB group), injection of BMCs with 50 µg control IgG (Sigma-Aldrich; IgG group), and injection of PBS only (CON group). BMCs were suspended in 200 µl of PBS and intramyocardial injection was performed into 2 sites (100 µl each) of the LV free wall, targeting the border areas [3].

To optimize the antibody dose, 1×10^7 ^BMCs were injected with 0, 10, 50, or 100 µg of anti-HMGB1 antibody in the same model (n≥3). At day 28, LVFAC was 31.5±1.2, 30.3±1.5, 26.6±1.3, and 26.4±1.8%, respectively. Then, 50 µg antibody was used in the main study.

### Detection of Released HMGB1

Peripheral blood was collected, from which serum was obtained by centrifugation. HMGB1 levels in the serum were determined in duplicate using a commercial ELISA kit (IBL international GMBH) according to the manufacture’s instruction.

### Histological Analysis

The hearts were excised, fixed with 4% paraformaldehyde, embedded in OCT compound, and quickly frozen in liquid nitrogen. Cryosections were cut and incubated with biotin conjugated Griffonia simplicifolia lectin I-isolectin B4 (1∶100, Vector), monoclonal anti-rat CD68 antibody (1∶100, AbD Serotec), monoclonal anti-rat CD86 antibody (1∶50, BD), monoclonal anti-rat CD163 antibody (1∶100, AbD Serotec), monoclonal anti-rat Ki-67 antibody (1∶50, DakoCytomation), and/or polyclonal anti-rat cardiac troponin-T (cTnT) antibody (1∶200, HyTest) followed by visualization using appropriate fluorophore-conjugated secondary antibodies (Molecular Probes). Samples were observed by a fluorescence microscopy (BZ8000, Keyence) with or without nuclear counter-staining using 4′, 6-diamidino-2-phenylindole (DAPI). Ten different fields were randomly selected in each border area of the samples and assessed. Another set of sections were stained with 0.1% picrosirius red, which enabled calculation of extracellular collagen volume fraction in border area by using NIH image-analysis software [3], [17], [22].

### Quantitative Analysis of Donor Cell Survival

Genomic DNA was extracted from the whole LV samples of female rats. To detect donor cell (male) survival, expression of the Y chromosome-specific *sry* gene in these samples was assessed by real-time polymerase chain reaction (PCR; Prism 7900HT, Applied Biosystems). The *sry* levels were normalized to the DNA amount using the autosomal single copy gene, oesteopontin. The number of surviving donor cells was estimated by correcting the relative *sry* expression using a standard curve as previously described [3], [17], [22].

### Measurements of Myocardial Gene Expression

Total RNA was extracted from the whole LV samples and assessed for myocardial expression of *IL-1β, TNF-α,* and *IL-10* by quantitative RT-PCR (Prism 7900HT, Applied Biosystems) as previously described [22]. TaqMan primers and probes were purchased from Applied Biosystems. Expression was normalized using *Ubiquitin C*.

### Statistical Analysis

All values are expressed as mean±SEM. Statistical comparison of the data was performed using the student’s unpaired *t*-test for the analysis of circulating HMGB1 levels. All other data were analyzed with one-way ANOVA followed by Fisher’s post-hoc analysis to compare groups. A value of *p*<0.05 was considered statistically significant.

## Results

### Poor Donor Cell Survival and Increased Extracellular HMGB1 After BMC Transplantation

**Figure 1:**
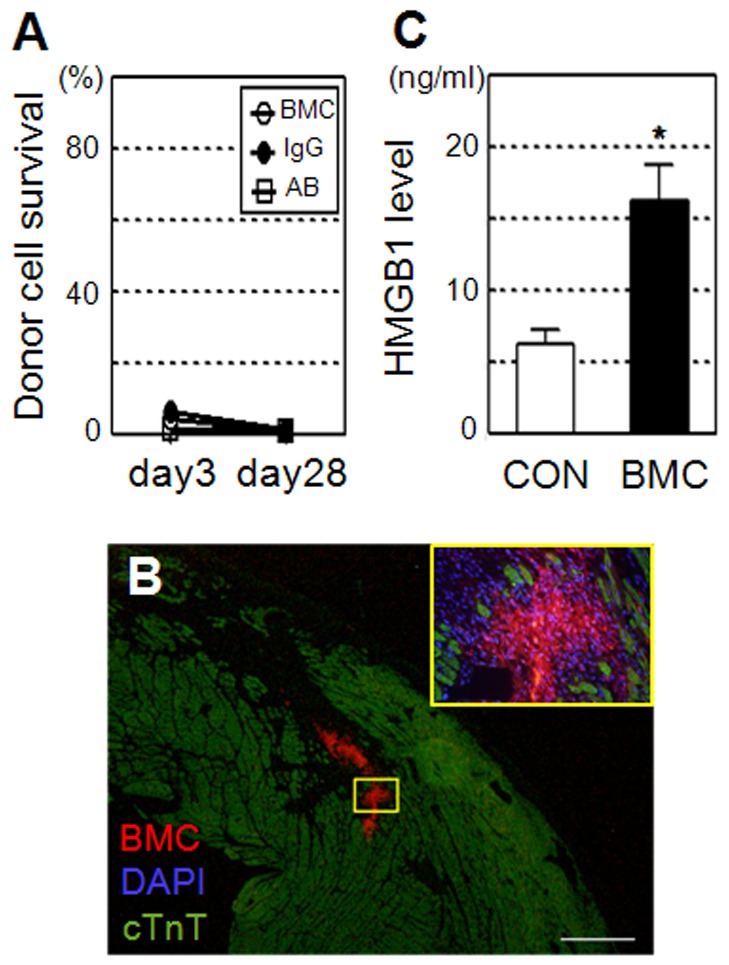
Poor donor cell survival and HMGB1 leakage after BMC transplantation. (**A**) Quantitative PCR for the male specific *sry* gene showed that the survival of male donor cells in female hearts was poor similarly in the BMC (BMC injection), IgG (BMC+control IgG injection), and AB (BMC+anti-HMGB1 antibody injection) groups at both days 3 and 28; n = 5∼7 in each point. (**B**) Clusters of DiI-labeled (red) donor BMCs were detected in the heart at day 3 after BMC transplantation. A higher magnification image of the yellow frame is shown. Green = cardiomyocytes (cTnT); blue = nuclei (DAPI). Scale bar = 300 µm. (**C**) ELISA showed that the circulating HMGB1 level was increased at 1 hour in the BMC group compared to the PBS injection control (CON group). *:*p*<0.05 *versus* the CON group, mean±SEM for n = 5 each.

Female rats suffering ischemic cardiomyopathy were randomly assigned to 4 groups; intramyocardial injection of syngeneic male BMCs (BMC group), intramyocardial injection of male BMCs with anti-HMGB1 neutralizing antibody (AB group), intramyocardial injection of male BMCs with control IgG (IgG group), and intramyocardial injection of PBS only (CON group). After each treatment, quantitative PCR for the male-specific *sry* gene demonstrated that donor cell survival after BMC transplantation was poor similarly in the BMC, AB, and IgG groups; below 10% at day 3, further decreasing to below 1% by day 28 (**Figure 1A**). Histological analysis detected islet-like clusters of donor cells at day 3 after BMC transplantation (**Figure 1B**). ELISA showed that the circulating extracellular HMGB1 level was 2.5-fold elevated one hour after BMC transplantation, compared to the PBS injection control (**Figure 1C**).

### Abolished BMC Transplantation-induced Cardiac Function Recovery by HMGB1-inhibition

**Figure 2:**
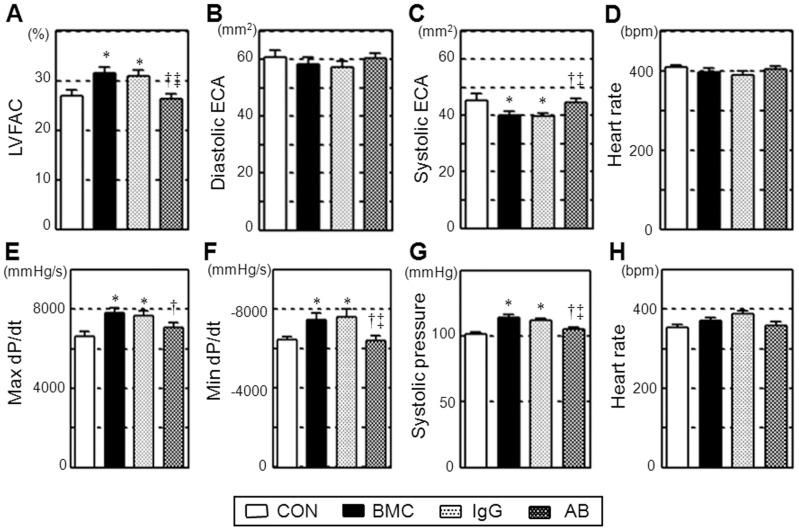
Abolished BMC transplantation-induced cardiac function recovery by HMGB1-inhibition. Cardiac parameters were measured by echocardiography (**A–D**) and catheterization (**E–H**) on day 28 after each treatment. Cardiac function was improved by BMC transplantation (BMC group) compared to the PBS injection control (CON group), while this effect was eliminated by antibody neutralization of HMGB1 (AB group), but not by control IgG administration (IgG group). LVFAC, left ventricular fractional area change; ECA, endocardial area. *:*p*<0.05 *versus* the CON group, ^†^:*p*<0.05 *versus* the BMC group. ^‡^:*p*<0.05 *versus* the IgG group, mean±SEM for n = 8∼10 in each group.

Four weeks after BMC transplantation (BMC group), echocardiography and cardiac catheterization consistently demonstrated that both systolic and diastolic LV function, in terms of LV fractional area change, max and min dP/dt, and systolic pressure, was improved compared to the control (CON group; **Figure 2**). Enlargement of LV systolic endocardial area in the control group was attenuated by BMC transplantation. Of note, these effects were largely abolished by HMGB1-inhibition (AB group), but not by control IgG administration (IgG group), indicating an important role of extracellular HMGB1 in the therapeutic benefits of BMC transplantation.

### Elimination of BMC Transplantation-induced Tissue Recovery by HMGB1-inhibition

**Figure 3:**
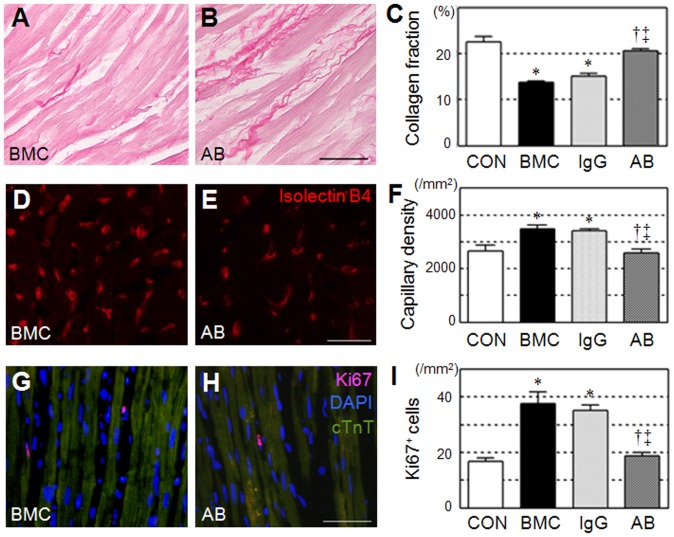
Eliminated BMC transplantation-induced tissue recovery by HMGB1-inhibition. Reduced extracellular collagen deposition (**A–C;** picrosirius red = red), increased capillary density (**D–F;** Isolectin B4 = red), and increased proliferation (**G–I;** Ki67 = red; nuclei = blue; cTnT = green) were observed in the border areas at day 28 after BMC transplantation (BMC group), compared to the PBS control (CON group). These effects were all abolished by anti-HMGB1 antibody neutralization (AB group), but not by control IgG administration (IgG group). Representative images of only BMC and AB groups are present (see **Figure S2** for additional images). Scale bars = 50 µm in **A, B, G, H** and 30 µm in **D, E**. *:*p*<0.05 *versus* the CON group, ^†^:*p*<0.05 *versus* the BMC group, ^‡^:*p*<0.05 *versus* the IgG group, mean±SEM for n = 5∼7 in each group.

To investigate the mechanism by which extracellular HMGB1 released in BMC transplantation improved post-MI cardiac function, we performed a set of histological studies with a focus on the paracrine effect. Consistent to previous reports [3], BMC transplantation (BMC group) attenuated post-MI pathological fibrosis, improved neovascular formation, and increased proliferation activity in the border areas at day 28, compared to the control (CON group; **Figure 3** **and Figure S2**). All these paracrine effects were, however, eliminated by HMGB1-inhibition (AB group), but not by IgG administration (IgG group), corresponding to the cardiac function change as shown in Figure 2.

### Modulation of M2/M1 Macrophage Polarization by BMC Transplantation through HMGB1

**Figure 4:**
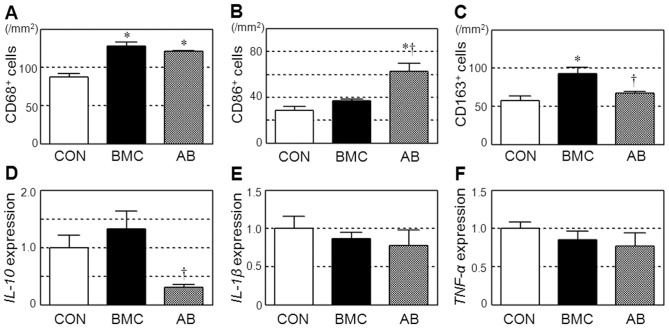
Modulation of innate immunity by BMC transplantation via released HMGB1. Accumulation of CD68^+^ pan-macrophages (**A**), CD86^+^ classically-activated pro-inflammatory M1 macrophages (**B**), and CD163^+^ alternatively-activated anti-inflammatory M2 macrophages (**C**) in the border areas at day 3 after each treatment was assessed by immunolabeling. See **Figure S3** for representative images. Myocardial expression of *IL-10* (**D**), *IL-1β* (**E**)), and *TNF-α* (**F**) at day 3 after each treatment was measured by quantitative RT-PCR. *:*p*<0.05 *versus* the CON group, ^†^:*p*<0.05 *versus* the BMC group, mean±SEM for n = 5∼7 in each group.

Additional immunolabeling showed that BMC transplantation increased myocardial accumulation of CD68^+^ pan-macrophages compared to the control (**Figure 4A** **and Figure S3A, B**). Here, the increase in CD86^+^ classically-activated pro-inflammatory (M1) macrophages was trivial (**Figure 4B** **and Figure S3D, E**), while the enhancement of CD163^+^ alternatively-activated (M2) macrophages was more obvious (**Figure 4C** **and Figure S3G, H**). As a result, the ratio of M2/M1 macrophage in the BMC group (92.9/36.9 = 2.52) was increased from 56.3/28.4 = 1.98 in the CON group. Of note, HMGB1-inhibition abolished the enhancement of CD163^+^ M2 macrophages (**Figure 4C** **and Figure S3I**) and exacerbated the increase in CD86^+^ M1 macrophages (**Figure 4B** **and Figure S3F**), thus largely reducing the M2/M1 ratio to 67.6/62.9 = 1.07. These results suggest that the HMGB1-mediated shift of macrophage polarization towards anti-inflammatory M2 macrophages might play a role in the BMC transplantation-induced myocardial recovery.

Quantitative RT-PCR showed that myocardial expression of the anti-inflammatory cytokine, *IL-10*, tended to be elevated by BMC transplantation compared to the control, while this was totally eliminated by inhibiting HMGB1 (**Figure 4D**). IL-10 is known to be secreted by alternatively activated M2 macrophages and also by Th2 cells that induce M2 macrophage differentiation [9], [25], [26]. The expression of *IL-1β* or *TNF-α* was not affected by either BMC transplantation or HMGB1-inhibition (**Figure 4E, F**).

## Discussion

Using a post-MI ischemic cardiomyopathy model in rat, we demonstrated that the BMC transplantation-mediated benefits, including increased neovascular formation, reduced collagen deposition, increased proliferation activity, favorable modulation of macrophage polarization, and resultant improvement of cardiac function, were all eliminated by antibody-neutralization of HMGB1. These data suggest that extracellular HMGB1 plays a role in the effects of BMC transplantation to recover the failing myocardium undergoing post-MI adverse remodeling and to improve global cardiac function. This finding is validated by the consistency with the previous report demonstrating that administration of recombinant HMGB1 protein achieved the same benefits as the BMC transplantation-mediated effects using the similar ischemic heart failure model in rats [17].

In view of the origin of extracellular HMGB1 occurring after BMC transplantation into post MI chronic heart failure, there are 4 theoretically possible sources: (i) passive release from dead donor BMCs, (ii) passive release from dead host (endogenous) cells, (iii) active secretion from surviving donor BMCs, and (iv) active secretion from host (endogenous) cells. It is likely that the major origin may be (i) passive release from dead donor BMCs, given the following information. [I] Survival of donor BMCs was largely limited, indicating that there was a considerable amount of donor cell death; [II] In this model of ischemic cardiomyopathy, death/damage of host cells in the heart and other organs by BMC injection is unlikely to be substantial; [III] There was an increased level of circulating HMGB1 as early as 1 hour after BMC injection. This time course is too rapid for inflammatory cells to actively secrete HMGB1 *via* transcription after stimulation [9], [27]. Having said these, we could not eliminate the possibility that extracellular HMGB1 from other sources, particularly from endogenous sources (host cells), might contribute to the paracrine effect of BMC transplantation. Experiments using HMGB1-deficient BMCs, either by knockout or knockdown, as donor would provide useful information to this point. However, HMGB1 knockout mice die immediately after birth [28], while on the other hand reproducible and satisfactory knockdown in primary rat unfractionated BMCs has not been established.

The role of extracellular HMGB1 to attenuate myocardial damage and to induce recovery and/or regeneration remains controversial [13]–[20]. This discrepancy may be relevant to different types of HMGB1 and different conditions of the host myocardium. In the settings of acute MI (without cell transplantation), a large amount of HMGB1 is actively secreted from accumulated inflammatory cells in addition to HMGB1 passively released from a large number of dead host cardiac cells. The dynamics and functions of these different types of HMGB1 are likely to be distinct, due to different phosphorylation, acetylation, and formation of complexes by binding other pathogenic molecules [9], [11]. Delicate balance between these types of extracellular HMGB1 may affect the overall effect, whether beneficial or harmful, of HMGB1. Our study therefore used a post-MI ischemic cardiomyopathy model to exclude these contaminating factors. The degree of HMGB1 both from inflammatory cells and dead host cardiac cells in this model is presumed to be much less than that in acute MI settings, and the frequency of host cardiac cell death by BMC injection is also negligible, compared to donor cell death.

It will be interesting to investigate whether the present results in unfractionated BMCs are applicable to other types of donor cells. There are published data in other cell types that appear to be contradicting to our findings in BMCs at a glance. Ziebart *et al.* have reported that the persistence of donor cells contributes to the therapeutic effect of transplantation of endothelial progenitor cells by using the inducible suicide gene [29]. Laflamme *et al.* have demonstrated that increase of donor cell presence (thus reducing donor cell death) by the treatment with pro-survival factors enhances therapeutic effects of transplantation of embryonic stem cell-derived cardiomyocytes [30]. However, in contrast, in the case of unfractionated BMCs, Yeghiazarians *et al.* reported that ultrasound-guided injection of extract of dead BMCs by freeze-thaw cycles achieves the similar therapeutic effect to injection of living BMCs [7], supporting our findings. This cell type-dependent controversy may suggest that the impact of HMGB1 released from dead donor cells to recover the damaged myocardium could be diluted/hidden in the case of transplantation of “more proficient” cells such as endothelial progenitor cells and embryonic stem cell-derived cardiomyocytes that have more substantial abilities of beneficial differentiation, secretion, and/or contraction. In addition, the extent and/or types of donor cell death after transplantation may vary according to donor cell types, affecting the release of HMGB1. Differences in the condition of the host heart, *i.e.* acute MI *versus* post-MI ischemic cardiomyopathy, may also influence the impact of extracellular HMGB1 from donor cells. Further studies to elucidate the role of extracellular HMGB1 in different donor cell types in the same experimental setting are warranted.

Macrophages are an important player in the progress and recovery of post-MI adverse ventricular remodeling [31], [32]. It has also been shown that HMGB1 and receptor for advanced glycation endproducts signaling play a role in the macrophage-involved tissue repair mechanism in the peripheral nerve [33]. Recent research has shown that macrophages can be functionally polarized into classically-activated M1 (pro-inflammatory) or alternatively-activated M2 (anti-inflammation and tissue healing) phenotypes according to the environmental condition [9], [26]. Stimulation with IFN-γ or TNF-α drives the macrophages into the M1 phenotype, which is characterized by a strong pro-inflammatory ability. In contrast, exposure to IL-4 or IL-13 generates M2 macrophages, which attenuate inflammation and enhance tissue recovery and healing. Importance of this polarization balance in the repair of damaged organs, including the heart, has been reported [9], [26]. Our results here uncovered that BMC transplantation enhanced “beneficial” M2 macrophages in the heart, for which extracellular HMGB1 was responsible. Therefore, HMGB1-mediated modulation of the macrophage’s polarization towards the M2 phenotype might be a part of the mechanism by which BMC transplantation recovers the damaged myocardium and improves cardiac function. Further study should focus on elucidation of the molecular mechanism of extracellular HMGB1 to modulate the M1/M2 macrophage polarization, in a simpler model.

A limitation of this study may be that our conclusion is based on the antibody neutralization experiments, which might carry a risk (though unlikely) of unexpected artifacts, such as unpredictable actions of the immune complexes generated. Investigations using HMGB1-deficient cells, either by knockout or knockdown, could add useful validation of our results, but these were not practical due to technical limitations as discussed above. Nonetheless, the consistency with the previous evidence that administration of HMGB1 protein induces the same effects as the BMC transplantation [17] supports our results.

In summary, our results demonstrated that extracellular HMGB1, which is derived from dead donor cells at least in part, plays a role of the effect of BMC transplantation to recover the damaged tissue by favorably modulating innate immunity in heart failure. This novel proof-of-concept will imply an important clue to further understand and refine BMC transplantation therapy for heart failure.

## Supporting Information

Figure S1 **Characterisation of BMCs by flow cytometry analysis.** Flow cytometry analysis showed that 4.6±1.7%of collected rat BMCs were positive for CD34 and 75.5±4.3% were positive for CD45. A representative image is presented.
(TIF)

Figure S2 **Supplement to** **Figure 3** **; HMGB1-inhibition abolished myocardial recovery by BMC transplantation. A–D:** Representative images of picrosirius red staining. Scale bar = 50 µm. **E–H:** Representative images of islectin-B4 staining (red). Scale bar = 30 µm. **I–L:** Representative images of immunofluorescent labelling with Ki-67 (red); blue = nuclei (DAPI). Scale bar = 50 µm.
(TIF)

Figure S3 **Supplement to** **Figure 4** **; Inflammation was modulated by BMC transplantation through HMGB1.** Representative images of immunofluorescent labelling with CD68 (**A–C**), CD86 (**D–F**), and CD163 (**G–I**). Green is for each target molecule; blue for nuclei (DAPI). Scale bars = 50 µm.
(TIF)
