# Supplementary figures and images for: Extracellular High Mobility Group Box 1 Plays a Role in the Effect of Bone Marrow Mononuclear Cell Transplantation for Heart Failure
Source: PLoS One. 2013 Oct 18;8(10):e76908. doi: 10.1371/journal.pone.0076908 (PMC3799896; doi:10.1371/journal.pone.0076908)

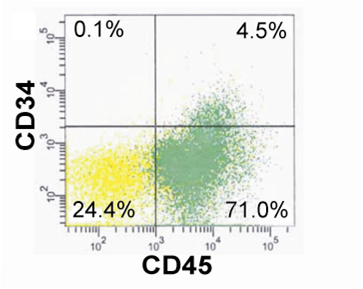

Supplement: Figure S1 — Characterisation of BMCs by flow cytometry analysis. Flow cytometry analysis showed that 4.6±1.7%of collected rat BMCs were positive for CD34 and 75.5±4.3% were positive for CD45. A representative image is presented. (TIF) [file pone.0076908.s001.tif]

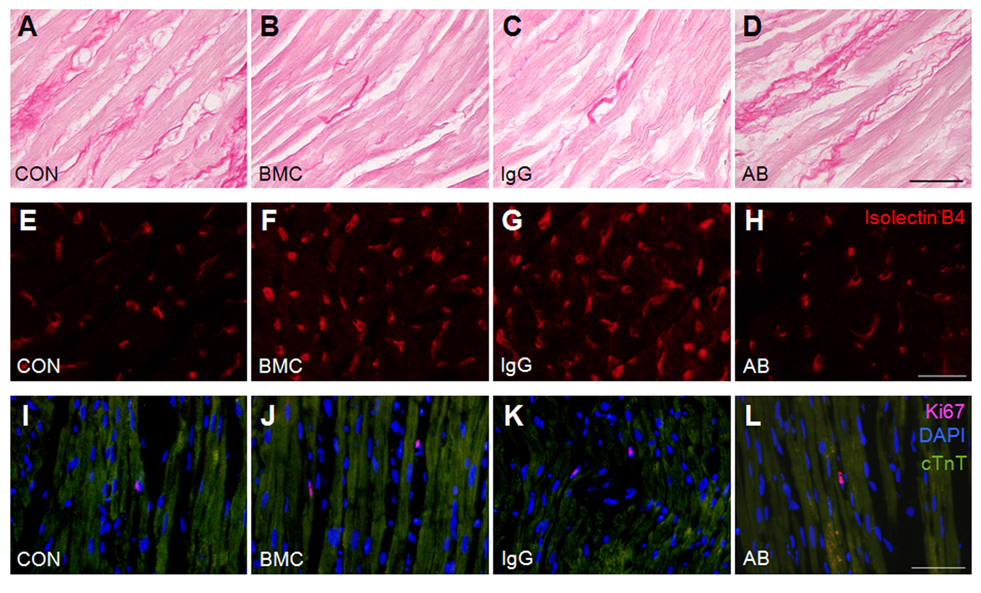

Supplement: Figure S2 — Supplement to Figure 3 ; HMGB1-inhibition abolished myocardial recovery by BMC transplantation. A–D: Representative images of picrosirius red staining. Scale bar = 50 µm. E–H: Representative images of islectin-B4 staining (red). Scale bar = 30 µm. I–L: Representative images of immunofluorescent labelling with Ki-67 (red); blue = nuclei (DAPI). Scale bar = 50 µm. (TIF) [file pone.0076908.s002.tif]

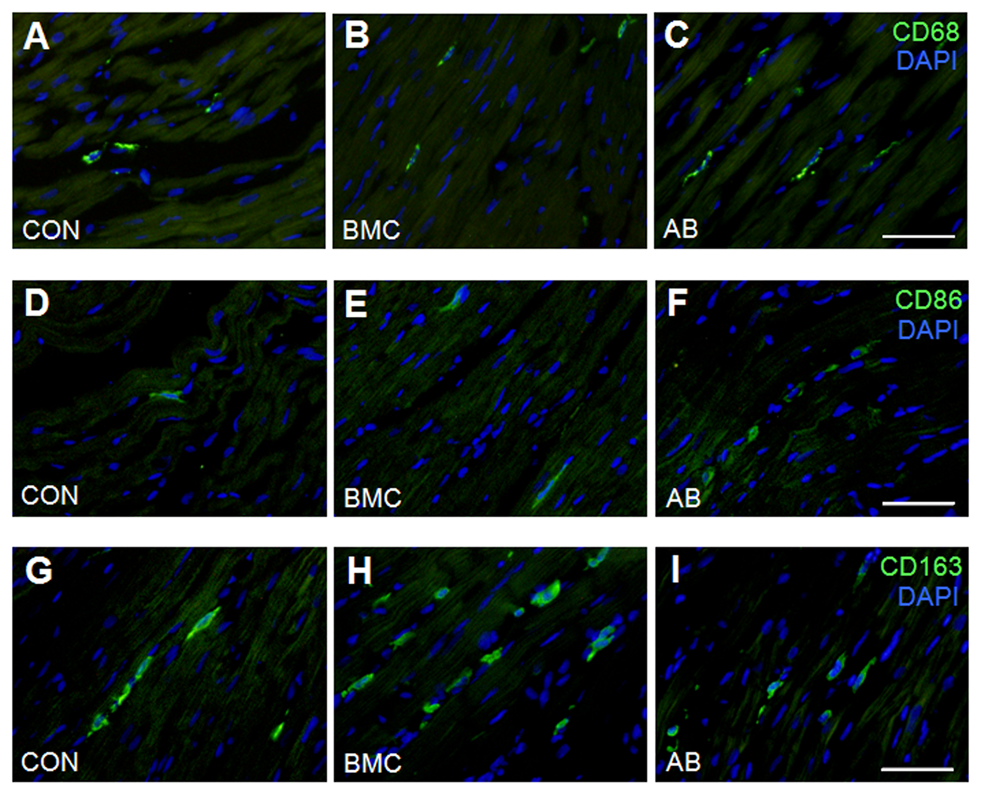

Supplement: Figure S3 — Supplement to Figure 4 ; Inflammation was modulated by BMC transplantation through HMGB1. Representative images of immunofluorescent labelling with CD68 (A–C), CD86 (D–F), and CD163 (G–I). Green is for each target molecule; blue for nuclei (DAPI). Scale bars = 50 µm. (TIF) [file pone.0076908.s003.tif]
